# Rapid identification and phylogenetic classification of diverse bacterial pathogens in a multiplexed hybridization assay targeting ribosomal RNA

**DOI:** 10.1038/s41598-019-40792-3

**Published:** 2019-03-14

**Authors:** Roby P. Bhattacharyya, Mark Walker, Rich Boykin, Sophie S. Son, Jamin Liu, Austin C. Hachey, Peijun Ma, Lidan Wu, Kyungyong Choi, Kaelyn C. Cummins, Maura Benson, Jennifer Skerry, Hyunryul Ryu, Sharon Y. Wong, Marcia B. Goldberg, Jongyoon Han, Virginia M. Pierce, Lisa A. Cosimi, Noam Shoresh, Jonathan Livny, Joseph Beechem, Deborah T. Hung

**Affiliations:** 1 Infectious Disease and Microbiome Program, Broad Institute of Harvard and MIT, 415 Main St, Cambridge, MA 02142 USA; 2 Infectious Diseases Division, Department of Medicine, Massachusetts General Hospital, 55 Fruit St, Boston, MA 02114 USA; 3 NanoString Technologies, Inc., 530 Fairview Ave N, Seattle, WA 98109 USA; 4 Present Address: UC Berkeley – UCSF Graduate Program in Bioengineering, Department of Bioengineering and Therapeutic Sciences, 1700 4th St, San Francisco, CA 94158 USA; 5 Present Address: Department of Chemistry, College of Arts & Sciences, University of Kentucky, Lexington, KY 40506 USA; 6 Present Address: NanoString Technologies, Inc., 530 Fairview Avenue N, Seattle, WA 98109 USA; 7 Research Laboratory of Electronics and Department of Electrical Engineering and Computer Science, Massachusetts Institute of Technology, 77 Massachusetts Ave, Cambridge, MA 02139 USA; 8 Infectious Diseases Division, Department of Medicine, Brigham and Women’s Hospital, 75 Francis St, Boston, MA 02115 USA; 9 Present Address: Baylor College of Medicine, 1 Baylor Plaza, Houston, TX 77030 USA; 10 Microbiology Laboratory, Department of Pathology, Massachusetts General Hospital, 55 Fruit St, Boston, MA 02114 USA; 11 Present Address: Twoporeguys, Inc., 2155 Delaware Ave, Santa Cruz, CA 95060 USA; 12 Department of Biological Engineering, Massachusetts Institute of Technology, 77 Massachusetts Ave, Cambridge, MA 02139 USA; 13 Department of Genetics, Harvard Medical School, 77 Ave Louis Pasteur, Boston, MA 02115 USA; 14 Department of Molecular Biology and Center for Computational and Integrative Biology, Massachusetts General Hospital, 185 Cambridge Street, Boston, MA 02114 USA

## Abstract

Rapid bacterial identification remains a critical challenge in infectious disease diagnostics. We developed a novel molecular approach to detect and identify a wide diversity of bacterial pathogens in a single, simple assay, exploiting the conservation, abundance, and rich phylogenetic content of ribosomal RNA in a rapid fluorescent hybridization assay that requires no amplification or enzymology. Of 117 isolates from 64 species across 4 phyla, this assay identified bacteria with >89% accuracy at the species level and 100% accuracy at the family level, enabling all critical clinical distinctions. In pilot studies on primary clinical specimens, including sputum, blood cultures, and pus, bacteria from 5 different phyla were identified.

## Introduction

After long relying on decades-old culture methods and traditional biochemical assays, modern clinical microbiology laboratories have begun to incorporate novel methods for bacterial pathogen identification in recent years^1^. Fundamentally, the challenge remains to recognize key distinguishing characteristics of a pathogen from a variety of clinical specimens as rapidly, sensitively, accurately, and inexpensively as possible. In recent years, several different molecular approaches have been brought to bear on this problem. For relatively pure samples of bacteria, mass spectrometry of cells or cell extracts provides a robust and rapid method for identification of a wide range of pre-specified bacteria with an assay time of a few hours^2^, though it works most robustly from positive colony growth on subcultured plates, which requires a longer processing time^3^. For a limited subset of pathogens, targeted DNA amplification-based methods, including panels for identification of common bacteria from cerebrospinal fluid^4^, respiratory specimens^5^, blood culture^6–8^, and recently even uncultured blood^9,10^, are in various stages of application. The genomics era brought the potential for dramatic advances in infectious disease diagnostics, led by whole-genome sequencing, which offers the most unbiased pathogen identification. However, to date, sequencing assays remain prohibitively costly, slow, and technically complex to perform and interpret^11^, and suffer from low signal-to-noise on primary clinical specimens, with host dominating over rare pathogen sequences^11–13^. Here we describe an applied genomics method that aims to recapitulate the phylogenetic breadth of unbiased sequencing in a simplified hybridization-based assay, capitalizing on the recent explosion of bacterial sequencing data in a format that requires no enzymology and thus can be performed directly and rapidly on clinical specimens.

Ribosomal RNA (rRNA) has long been a target of interest in bacterial identification. As a structural and enzymatic component of core cellular machinery, the 16S and 23S rRNA genes are present in all bacteria, and their sequences are exquisitely conserved within a species. They therefore contain rich evolutionary information: sequence divergence amongst species at this locus reflects evolutionary distance^14–16^. These genes contain a characteristic pattern of constant (across species) and variable (between species) regions^15,17,18^ that allow for amplification of the species-informative variable subsections of the genes from diverse bacteria using degenerate primers to the conserved regions^19,20^. Such amplicon sequencing, often from DNA encoding the 16S rRNA subunit, is utilized for bacterial characterization in microbiome studies^21,22^ and occasionally for species identification in clinical diagnostics when standard microbiological methods fail^23,24^. In addition, rRNAs are by far the most abundant RNA species in a bacterium; at >10,000 copies per cell^25–27^, they typically comprise >85% of the RNA mass of a cell^28,29^ and thus serve as an abundant target for direct characterization even in the absence of amplification.

Taking advantage of these features of rRNA, we designed and developed an approach for broad-range bacterial identification through multiplexed hybridization of fluorescent DNA probes to ribosomal RNA, with the goal of matching the advantages of unbiased sequence-based pathogen identification in a simplified, more rapid applied genomics assay that requires no amplification or enzymology. This approach leverages the NanoString technology platform, which enables the simultaneous sensitive, quantitative, multiplexed measurement of hundreds of different RNA molecules in crude samples through hybridization of a pool of fluorescently barcoded bipartite 50mer oligonucleotide probes to the target RNA molecules in a crude sample, capture of these probe-target complexes, and enumeration through fluorescence microscopy^30^. While we^31^ and others^32^ have previously used NanoString to detect species-specific mRNA targets for identification of specific pathogens, rRNA offers a more universal and far more abundant target, enabling greater assay breadth and sensitivity. We previously demonstrated that NanoString assays can identify a limited set of bacterial rRNA targets with high sensitivity^33^; here, we sought to take on the challenge of designing a probeset that would span the breadth and complexity of phylogenetic diversity encompassed by clinically relevant bacterial pathogens, thereby detecting and identifying a wide range of bacterial species in a single, simple assay. Such pan-phylogenetic rRNA-directed hybridization probesets have been attempted using microarrays, but the high degree of similarity between rRNA targets in closely related species prevented broad implementation^34,35^. We revisited this goal, aided by a more quantitative RNA detection platform and informed by the wealth of available bacterial genomes that enabled us to consider sequences from thousands of different bacterial species in designing hybridization probes with predicted specificity at varying taxonomic levels. To this end, we designed 180 pairs of hybridization probes that would recognize and distinguish the 16S and 23S ribosomal RNAs from 98 clinically relevant bacterial pathogens (Table S1), including probe pairs targeting regions that are highly species-specific as well as others that target conserved regions within every level of the taxonomic hierarchy (genus, family, order, class, phylum) (Supplementary Fig. S1; Table S2). We call this panel of 180 probe pairs the phylogeny-informed rRNA-based strain identification (Phirst-ID) probeset.

Bacterial species are identified by integrating data from all probes in the Phirst-ID probeset. Because of the extreme sequence conservation at these loci, often varying by only a few nucleotides between closely related species, few probes hybridize in a binary manner with only the intended target(s). Thus, unlike typical quantitative hybridization-based assays such as microarrays or mRNA-directed NanoString assays in which signal intensity reflects abundance of the target, here, signal intensity instead reflects the hybridization efficiency of the probe pair for different targets, governed by the degree of sequence complementarity between each probe pair and its target. (This assumes that the 16S and 23S rRNA targets are in fixed relative abundance.) Bacterial identity is thus inferred from the ensemble pattern of reactivity across the 180 probe pairs with the target bacterial rRNA. This nuanced interpretation of hybridization efficiency as a proxy for sequence relies on the accurate, quantitative nature and broad linear dynamic range of the NanoString assay platform, as well as its ability to multiplex across hundreds of targets in a single reaction^30^, offering advantages over prior efforts at rRNA-directed, hybridization-based pathogen identification using microarrays^35–37^ or fluorescence *in situ* hybridization^25^.

This multiplexed hybridization strategy has several advantages over other methods currently in use or under development. First, this approach queries a far broader range of pathogens in a single assay than can be assessed using multiplexed amplification assays and thus does not require foreknowledge of the bacterial species, at least for those contained in the large set of targeted pathogens, or their close relatives. Second, this assay measures the rRNA itself, rather than the DNA gene that encodes it, thus capitalizing on the intrinsic amplification of the highly abundant targets and increasing its utility for samples with low pathogen burdens. Third, unlike enzymatic amplification or mass spectrometry, hybridization is both highly specific to its target sequence and robust to considerable variation in sample composition. Thus, this approach can be deployed on crude lysate preparations from diverse sample types, including primary clinical samples with a vast excess of host background that often confounds other agnostic identification methods such as sequencing or mass spectrometry. Together, these features enable more rapid answers in a clinical setting; this assay is capable of returning answers on a primary clinical specimen in 3 hours.

Using the Phirst-ID probeset, we tested 117 isolates from 64 distinct species of bacterial pathogens across a wide range of bacterial phylogeny (29 genera, 20 families, 15 orders, 8 classes, 4 phyla) to create a reference set of probeset reactivity patterns (PSRPs) against known species. To determine the identity of unknown samples, we developed a simple classification scheme based on the Pearson correlation between the PSRP of a test sample and the PSRPs of known bacterial species in this reference set. The identity of the test sample could be assigned based on the known bacterial species with which it had the highest Pearson correlation. Importantly, these Pearson correlations of PSRPs reflect known phylogenetic relationships across this diverse range of bacteria. We illustrate the robustness of the hybridization-based assay by performing pathogen detection without amplification directly from clinical samples of sputum, cultured blood, and pus.

## Results

### Pathogen selection and Phirst-ID probeset design

Aiming to target a comprehensive, if not exhaustive, list of human bacterial pathogens for identification, we selected 98 priority bacterial pathogens across 40 genera, 28 families, 21 orders, 11 classes, and 6 phyla as targets for rRNA probe design (Table S1), as well as 1122 other bacterial species to serve as additional outgroups and/or in-groups at higher phylogenetic levels, for a total of 1220 bacterial species. We first set out to design species-specific probes for the 98 targeted pathogens. We identified 16S and 23S regions that were conserved within a species but that were the most variable between species, with the goal of uniquely recognizing as many of the 98 target pathogens as possible at the species level, considering all other 1219 species as outgroups. The extreme conservation of rRNA sequences limited us to 68 probe pairs designed to have some species-level discrimination for the species they were designed to recognize, though the majority of these were suspected to have some degree of cross-reactivity with other closely related species. To identify rRNA regions that were most conserved within a genus but that were the most variable and thus discriminatory between genera, we next performed the same type of analysis at the genus level, again including the full set of 1220 bacterial species (Supplementary Fig. S2). In order to ultimately design one or more probe pairs that would recognize all members of each targeted genus, family, order, class, and phylum without recognizing any species excluded from that taxonomic classification, this process was performed iteratively for each of the higher taxonomic levels for each targeted pathogen.

The final Phirst-ID probeset contained 180 total probe pairs: 68 predicted to be specific at the species level, 50 at the genus level, 1 at the family level, 33 at the order level, 17 at the class level, and 11 at the phylum level (Supplementary Fig. S1; Table S2). The inclusion of probes at progressively higher phylogenetic levels served two purposes. First, we expected they would improve accuracy in identifying targeted species through multiple recognition events that enable classification at multiple phylogenetic levels, thus increasing confidence in the identification. Second, and perhaps more importantly, their inclusion would allow the recognition and limited characterization of bacteria not targeted for probe design in our initial list of 98 pathogens; as long as a bacterial species falls in or near an area of the phylogenetic tree covered by the Phirst-ID probeset, we anticipated that probes at some phylogenetic level would recognize it, and thus detect and partially characterize it, providing clinically relevant information even if not enabling identification at the species level. Collectively, these probes target a wide swath of the 16S and 23S rRNA subunits (Supplementary Fig. S1).

### Diverse bacterial species yield unique probeset reactivity patterns

To assess the reproducibility of the multiplexed, hybridization-based assay itself, we determined the similarity between technical and biological replicates of identical strains. As expected for the NanoString assay format^30^, technical replicates, i.e., separate aliquots from an identical lysate preparation, showed near-perfect correlation (*R* > 0.997; Supplementary Fig. S3a). Biological replicates, i.e., different lysate preparations of the same strain, showed similarly strong correlations (*R* > 0.990; Supplementary Fig. S3b).

**Figure 1:**
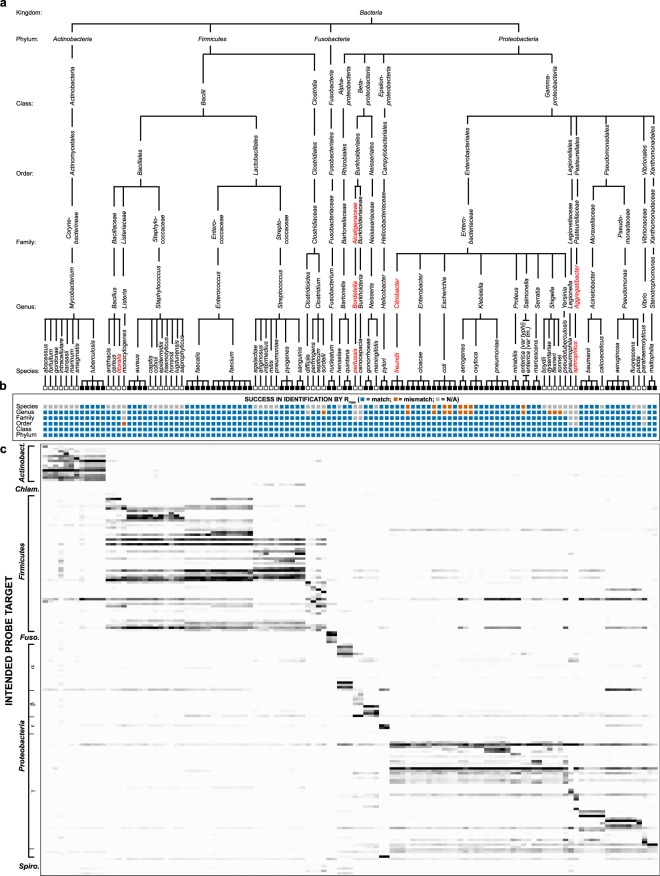
Normalized signal intensity of 180 Phirst-ID probes against a reference set of 117 strains. (**a**) Taxonomic classifications of the 117 strains tested (top). Black boxes indicate species for which multiple isolates were tested; white boxes indicate species for which a single isolate was available. Taxonomic levels not targeted in probeset design are shown in red. (Note: distances on the taxonomic diagram do not represent phylogenetic distances.) (**b**) Accuracy of identification at each taxonomic level based on highest non-self Pearson correlation coefficient (*R*_max_). Blue boxes indicate correct identification; orange boxes indicate mismatch; gray boxes indicate cases where no non-self match is possible within the test set. (**c**) Normalized intensity data from 180 probes displayed as a heatmap. Probes are displayed in the order listed in Table S2, with broad categories of intended targets indicated at left.

To validate the Phirst-ID probeset, we tested its ability to distinguish different clinically relevant pathogens of interest. We obtained samples of 64 different bacterial species, including 60 from the original list of 98 targeted species, plus 4 additional species for which the probes were not explicitly designed, encompassing a wide range of bacterial diversity (Table S3; Fig. 1). For 22 species, we obtained more than one isolate (i.e., biological replicates). In all, this reference set included 117 distinct strains, largely clinical isolates. Quantitative hybridization data are shown in Fig. 1. Each broad category of pathogens (mycobacteria, Gram positives, Gram negatives) qualitatively exhibited distinct Phirst-ID PSRPs that largely matched the phylogeny of the intended targets. However, these PSRPs were complex, and distinctions between individual closely-related species were more often subtle than binary; relatively few probes were truly species-specific in an all-or-none manner.

### Pearson correlations convey identity and phylogenetic characterization of test strains

**Figure 2:**
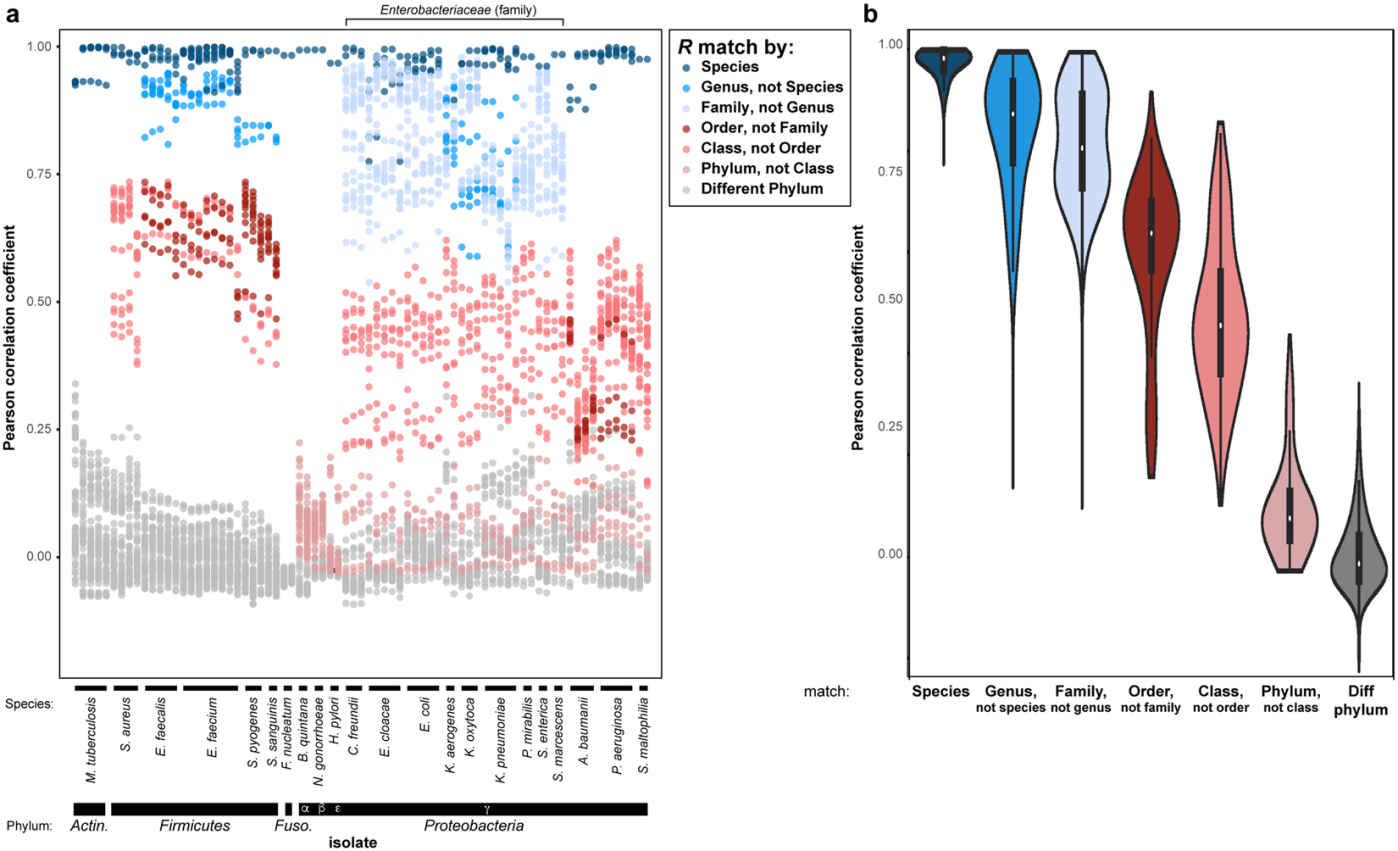
Probeset reactivity patterns (PSRPs) convey identity and correlate with phylogeny. (**a**) All 116 non-self pairwise Pearson correlation coefficients (*R*) plotted for each of the 75 isolates from 22 species tested in replicate, colored by taxonomic relationship of the paired species. Order of species is the same as Fig. 1a (black boxes only). (**b**) Distributions of pairwise *R* values for species pairs at each indicated taxonomic relationship. Data from same species reflects only the isolates shown in (**a**); all other data reflects all 117 isolates (including species for which single isolates were available).

We aimed to implement a classification scheme to identify individual bacterial species using this rich reference dataset. Based on the qualitative differences seen in PSRPs between strains, we hypothesized that Pearson correlations of the set of signal intensities for all probes would reflect phylogenetic relatedness between pairs of strains, with the highest correlations found for members of the same species. Pearson correlations represent a simple analytic method for incorporating information from all probes in a sample, intrinsically giving high weight to those that react the most strongly with a given species – a desirable feature, since probes with high signal are most complementary to the rRNA of the strain being tested and thus encode the most specific sequence information about that strain. We therefore computed pairwise Pearson correlation coefficients for all possible pairs of the 117 reference strains (Supplementary Fig. S4). Pairwise correlation coefficients are indeed highest for isolates of the same species (mean 0.98, standard deviation 0.02), progressively lower for those of the same genus, family, class, and phylum, and lowest for those of distinct phyla (Fig. 2).

We tested the hypothesis that the highest non-self Pearson correlation (*R*_max_) in the reference panel would identify the correct phylogeny of an unknown bacterium, from phylum through species. We used a “leave-one-out” analysis in which each of the 117 strains in the test set was compared with the other 116. At the species level, this approach was only possible for the 75 samples comprising 22 species for which we could test more than one isolate; for the remaining 42 species for which we obtained only one isolate, that strain could not be matched to itself using a leave-one-out approach. For 75 isolates amenable to this approach, the *R*_max_ identified the correct species in 67 of 75 cases (89.3%; Fig. 1b, Supplementary Fig. S5a). Similarly, of 109 isolates across 21 genera for which we obtained more than one isolate, the highest non-self *R*_max_ identified the correct genus in 97 of 109 (89.0%; Fig. 1b, Supplementary Fig. S5b). Accuracy improved at higher taxonomic levels (Fig. 1b, Supplementary Fig. S5c–f), as the highest non-self *R*_max_ was nearly perfect in identifying family (111/111 correct; 100%), order (113/114, 99.1%, mispairing *Listeria monocytogenes* with the *Lactobacillales* order rather than *Bacillales*), class (117/117; 100%), and phylum (117/117; 100%).

All eight species-level and 11 of 12 genus-level misclassifications were mispairings within the *Enterobacteriaceae* family of enteric Gram negative rods (GNRs), which are the set of species most similar to each other by rRNA sequence amongst those we tested. Three of the eight species-level and six of the 12 genus-level misclassifications involved mispairings between *Escherichia coli* and *Shigella* isolates; these genera are particularly similar in rRNA and even core genome sequence, leading some to argue that they should be considered the same species^38–40^. Indeed, species distinctions within this closely-related family are somewhat fluid, with *Klebsiella aerogenes* having recently been reassigned from the *Enterobacter* genus; an additional two genus and two species-level mismatches involved this species. The only genus-level mispairing outside the *Enterobacteriaceae* family involved another recently reassigned species, as the sole isolate of *Clostridium sordelii* was paired with *Clostridioides difficile*, which until recently was considered a member of the *Clostridium* genus, rather than with one of the two other *Clostridium* species in the reference set. These readily explicable cases highlight the current fluidity and challenges for bacterial taxonomy in the era of genomics, with little practical consequence in clinical practice, since the *Enterobacteriaceae* share similar clinical characteristics. Aside from these cases, the only mispairing occurred for *Listeria monocytogenes*, a species for which we only had a single isolate from its family, the *Listeriaceae*, in the reference set. A larger, more comprehensive reference set would be expected to improve the identification of cases such as *C. sordelii* and *L. monocytogenes*.

Beyond species identification by the highest *R*_max_, the overall pattern of all pairwise Pearson correlations from this probeset reflects the known phylogenetic relationships between the organisms remarkably well, both for species for which multiple isolates were tested (Fig. 2a) and even for species for which only a single isolate was tested (Supplementary Fig. S6). These findings suggest that, even when a new species is encountered against which the probeset has not been validated, the *R*_max_ is likely to be highest for the closest phylogenetic match among species in the reference set (Supplementary Fig. S6). Aggregating these data from all tested strains confirms this progressive trend, with values declining and distributions widening for correlations between more phylogenetically distant isolates (Fig. 2b).

### Phirst-ID probeset is able to distinguish clinically relevant pathogens

**Figure 3:**
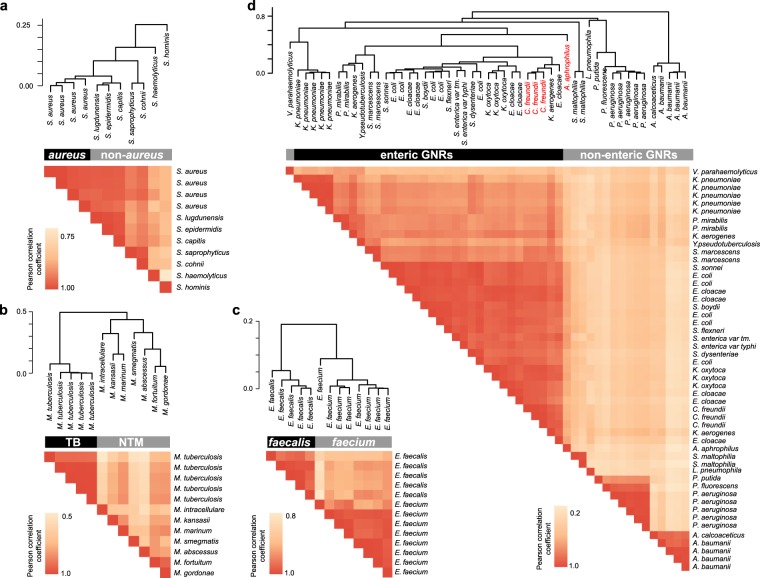
Hierarchical clustering of strains based on PSRPs identifies clinically relevant pathogen subsets. Pearson correlation coefficients (*R*) for (**a**) *Staphylococcus aureus* vs non-*aureus* species; (**b**) *Mycobacterium tuberculosis* vs non-tuberculous mycobacteria; (**c**) *Enterococcus faecalis* vs. *E. faecium*; (**d**) *Enterobacteriaceae* vs non-enteric GNRs. Species not targeted in probeset design are labeled in red. Trees above reflect distances based upon unsupervised hierarchical clustering of (1-*R*), with the indicated scale at top left. Heatmaps below display each pairwise *R* value, with color scale at lower left. Strain order is the same on horizontal and vertical axes; self-correlations are included on the diagonal.

In clinical practice, identification at the species level is more critical for some bacterial pathogens than others, either for prognostication or for informing empiric antibiotic selection. *Staphylococcus aureus*, for instance, is one of the most common causes of serious bloodstream infections, whereas other species of *Staphylococcus* (collectively known as the coagulase-negative staphylococci in the clinical setting) that appear identical on Gram stain, are often contaminants, or less virulent true pathogens. For this clinically important case, Phirst-ID correctly identified four independent clinical isolates of *S. aureus* by *R*_max_; i.e., each was most similar to another *S. aureus*, rather than to any of seven common coagulase negative staphylococcal species in the reference set. Building confidence in this phylogenetic approach to pathogen identification, unsupervised hierarchical clustering based on Pearson correlation coefficients from the 11 *Staphylococcus* strains from the reference set demonstrated that the four *S. aureus* strains clustered together, apart from the seven non-*aureus* strains, underscoring the ability of this probeset to make this critical distinction (Fig. 3a). Similarly, *Mycobacterium tuberculosis* in a clinical sample carries far different implications for both patient outcomes and infection control measures than non-tuberculous mycobacteria, though they are indistinguishable by acid-fast stain. One common laboratory strain and four clinical isolates of *M. tuberculosis* were correctly identified, clearly clustering apart from seven other mycobacterial species in the reference set (Fig. 3b). Finally, because *Enterococcus faecalis* and *E. faecium* exhibit dramatically different rates of resistance to both ampicillin and vancomycin^41,42^, identification to the species level is critical in informing empiric antibiotic choice. Five clinical isolates of *E. faecalis* were correctly identified, clustering independently and far from eight clinical isolates of *E. faecium* (Fig. 3c), again underscoring that the Phirst-ID probeset can make critical clinical distinctions.

The most important and useful clinical distinction to be made among the GNRs is between the enteric GNRs (defined by the single *Enterobacteriaceae* family), and the non-enteric GNRs (comprising a number of families including *Pseudomonas, Acinetobacter*, and *Stenotrophomonas* genera, among others) due to intrinsic differences in susceptibility to beta-lactam antibiotics that would dictate different empiric antibiotic choices: non-enteric GNRs require higher-generation, “anti-pseudomonal” penicillins or cephalosporins, and also have considerably lower barriers to resistance to carbapenems, than do enteric GNRs. While this probeset could not distinguish amongst all *Enterobacteriaceae* at the genus or species level, the 34 enteric GNR isolates tested, across 15 species, were clearly distinct from 17 isolates of six genera of non-enteric GNR pathogens (including 14 isolates from three commonly encountered genera *Acinetobacter*, *Pseudomonas*, and *Stenotrophomonas*, each of which were individually distinguishable) (Fig. 3d). In each case, while distinctions were made based on overall PSRP, each difference was driven in part by specific probes designed to be most reactive to individual species within the taxa in question (Supplementary Fig. S7).

### Classification accuracy for untargeted pathogens

Of the 64 species we tested, 60 were among the 98 species targeted for probe design. The four species that were not explicitly targeted in the design process allowed us to test how well the probeset performs on “untargeted” species for which it was not intentionally designed, an important feature of an unbiased diagnostic assay. Our hypothesis was that the bacterial phylogenetic diversity captured in this probeset would be sufficient to match the untargeted species with its nearest phylogenetic neighbor in the reference set, even if that neighbor were only matched quite distantly, thereby providing some useful characterization of the untargeted species. The four untargeted species were *Bacillus litoralis*, *Bordetella pertussis*, *Citrobacter freundii* (three isolates), and *Aggregatibacter aphrophilus;* in all four cases, the species in the reference set whose PSRP most correlated with that of the untargeted species was indeed a member of the closest possible taxonomic grouping (Supplementary Fig. S8a).

In the case of *B. litoralis*, we incorporated two other species in the *Bacillus* genus (*B. anthracis* and *B. cereus*) in design and testing. The PSRP of *B. litoralis* had an *R*_max_ of 0.932 with the PSRP of *B. cereus*. For *C. freundii*, we did not include any members of that genus in the design process, but did include many members of its family, the *Enterobacteriaceae* (8 other genera and 14 other species). The PSRPs of the three *C. freundii* isolates best correlated with each other despite not being included in the design process (*R*’s = 0.982–0.997); among designed species in the reference set, they best matched the PSRP for another species in the same *Enterobacteriaceae* family, *Klebsiella oxytoca* (*R*’s = 0.954–0.963). Finally, for both *B. pertussis* and *A. aphrophilus*, their nearest neighbors in the reference set were quite distant. For *B. pertussis*, the closest relative in both design and testing was *Burkholderia cenocepacia*; both are members of the *Burkholderiales* order but diverge at the family level. The PSRP of *B. pertussis* indeed matched *B. cenocepacia* most closely (*R*_max_ = 0.91). For *A. aphrophilus*, the two closest relatives included in the design process, *Haemophilus influenzae* and *Pasteurella multocida*, are in the same *Pasteurellaceae* family; however, we could not obtain samples of these two pathogens for testing. Thus, the closest relative tested was related only by class (*Gammaproteobacteria*), of which 52 isolates representing 6 orders, 7 families, 15 genera, and 24 species were tested. The PSRP of *A. aphrophilus* indeed correctly placed it in the *Gammaproteobacteria* class, albeit with worse correlations, consistent with its more distant relationship with any test strains. While its highest *R*_max_ was with *Salmonella enterica var. typhimurium* (*R* = 0.694), the probes with the highest signal intensity for the tested *A. aphrophilus* were probes designed for the matching *Pasteurellales* order and the closely related *Haemophilus* genus (Supplementary Fig. S8b); *A. aphrophilus* was formerly classified as a *Haemophilus* species.

### Pilot studies on clinical samples reveal causal pathogens

To test the potential clinical utility of this approach, we applied the Phirst-ID probeset to 15 consecutive sputum samples collected from patients at Brigham and Women’s Hospital that were confirmed by standard methods to be culture-positive, and to 37 consecutive positive blood cultures from unique patients at Massachusetts General Hospital. Sputum was chosen as a high-value clinical sample with moderate bacterial burden and relatively high host background. Blood culture was chosen due to the clinical importance of prompt diagnostic information in bacteremic patients. Clinical samples were run, blinded to the corresponding result obtained from the hospital clinical microbiology lab, which was unmasked for comparison only after completion and analysis of the multiplexed hybridization assay.

**Figure 4:**
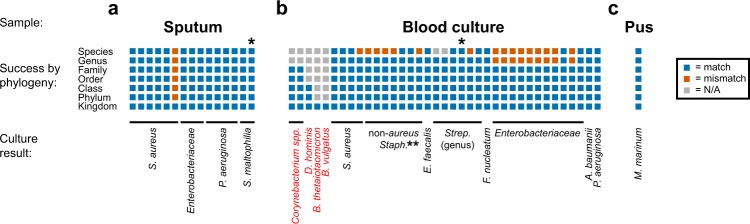
Probeset performance in identifying pathogens from clinical samples. Accuracy of identification at each phylogenetic level based on *R*_max_ compared with the reference set of 117 isolates for (**a**) sputum, (**b**) cultured blood, and (**c**) pus. Blue boxes indicate correct identification; orange boxes indicate mismatch; gray boxes indicate cases where no match is possible with the reference set at that taxonomic level. Each type of clinical sample is ordered by taxonomy of the pathogen identified by the clinical microbiology laboratory, listed below. Species not targeted in probeset design or included in the reference set are labeled in red. *Mixed sample, as described in main text. **All species-level errors amongst the non-aureus staphylococci involved mispairings with other non-aureus staphylococci, not with *S. aureus*.

For sputum samples, the strain from the reference set with the highest *R*_max_ was an exact species-level match to the species identified by the clinical microbiology laboratory in 14 of 15 cases (Fig. 4a). Among the correct matches, all but one Pearson correlation was >0.830 (Supplementary Fig. S9a), indicating good agreement at the species level. Importantly, despite differences in sample composition that can reduce the *R*_max_ compared to those of pure *in vitro* bacterial cultures (i.e., competing nucleic acids from either host background or other bacterial species that affect hybridization kinetics or equilibrium binding), a species could still clearly be identified in all but one case.

Working with primary clinical specimens provided the opportunity to begin to assess the increased complexity inherent to these sample types compared with isolates. Sputum in particular may include multiple pathogens, or a mixture of respiratory pathogen(s) and oral flora. One correctly identified sample containing *S. aureus* nonetheless had a lower *R*_max_ than the other cases (Supplementary Fig. S9a, sample 1). On examining its PSRP (Supplementary Fig. S9b), in addition to obvious signal from the expected staphylococcal probes, strong signal is also seen from probes designed to recognize the *Pasteurellales* order, the *Haemophilus* genus, and the *H. influenzae* species. Indeed, the sputum Gram stain revealed both Gram positive cocci in clusters (a morphology consistent with *Staphylococcus* species) and Gram negative rods, which failed to grow on culture but may have been the source of this additional signal we observed from uncultured sputum. The one failure to identify the cultured pathogen, *S. aureus* (Fig. 4a and Supplementary Fig. S9a, sample 6), occurred in a sample that showed abundant oral flora on Gram stain. While its PSRP showed clear reactivity from *S. aureus* probes that were well above background (Supplementary Fig. S9b), signal intensity and thus *R*_max_ in this specimen was dominated by probes from the *Actinobacteria* phylum and the *Actinomycetales* order, likely real signal from the oral bacteria in the sample. In a third sample that gave rise to a mixed culture containing *Stenotrophomonas maltophilia*, *Pseudomonas aeruginosa* and *Chryseobacterium indologenes*, the probeset signal was dominated by *S. maltophilia* (Supplementary Fig. S9a, sample 15) with low-level signal detected from *P. aeruginosa* probes, which were nonetheless well above background. Thus, even in obviously mixed samples, the Phirst-ID probeset was able to identify the dominant pathogen, though not surprisingly, it failed when the pathogen was in lower abundance than other flora.

Assays of 37 consecutive positive blood culture bottles from unique patients collected from Massachusetts General Hospital over a period of one week were positive for a surprisingly and unusually wide diversity of pathogens (21 distinct species across five phyla, including seven species not in our reference set. The *R*_max_ correctly distinguished critical clinical classes of pathogens in 24 of 25 cases (97.5%, including 13 of 13 *Enterobactericeae* vs. non-*Enterobactericeae*, 10 of 11 *S. aureus* vs. non-*aureus* staphylococci, and 1 of 1 *E. faecalis* vs *E. faecium*) and correctly identified the family for all 34 samples that had family-level matches in the reference set (Fig. 4b). It however struggled with the challenging albeit less clinically important distinctions that we identified previously, specifically with distinguishing among the different enteric GNRs and among the different coagulase negative staphylococci. Several relatively rare causes of bacteremia, including *Fusobacterium nucleatum* and two non-enteric GNRs with high rates of antibiotic resistance, *Pseudomonas aeruginosa* and *Acinetobacter baumanii*, were identified with confidence based on their unique PSRPs (Supplementary Fig. S9c). For samples collected in real-time, we obtained this clinically valuable information the same day the blood cultures signaled positive, one day faster than the standard workflow at the MGH microbiology lab, which relies on matrix-assisted laser desorption/ionization time-of-flight (MALDI-TOF) mass spectrometry signatures from subcultured colony growth for species identification.

These blood culture samples also provided several opportunities for assessing the Phirst-ID probeset’s ability to characterize untargeted pathogens that were absent from the reference set. In each of seven cases, the *R*_max_ identified a match at the closest possible taxonomic level. Two previously untested streptococci, *S. dysgalactiae* and *S. gallolyticus*, each matched *S. mitis* (*R*_max_ 0.96–0.97). Five other species were more distantly related to any strains targeted for design or in the reference set. Three were in the phylum *Actinobacteria*: two *Corynebacterium* species and a *Dermabacter hominis*, which each matched most closely with a *Mycobacterium* species (same family as *Corynebacterium*, same class as *Dermabacter*), albeit with lower *R*_max_ (<0.75) suggesting an imprecise pairing. The remaining two were of the phylum *Bacteroidetes*, which was not included in either design or the reference set. Yet both *Bacteroides thetaiotaomicron* and *B. vulgatus* were detected by a few probes such that at least the presence of a bacterium could be confirmed (Supplementary Fig. S9d). In addition, one blood culture initially identified by the clinical microbiology laboratory as *S. mitis/oralis* was later found to be a mixed culture that also contained two types of *Granulicatella spp*. PhirstID identified this only as *S. mitis*; of note, *Granulicatella* is a nutritionally variant genus that was until recently also classified as a *Streptococcus*.

The Phirst-ID probeset was also tested on a sample of pus obtained from the finger of a laboratory worker exposed to *Mycobacterium marinum*. Eight days after the exposure, the finger had become erythematous, indurated, and tender, with purulent drainage concerning for bacterial infection. Clinical suspicion was highest for either common skin and soft tissue pathogens, e.g. *Staphylococcus* or *Streptococcus* species, or *M. marinum* given the recent exposure. This distinction is difficult to make by conventional means, since *M. marinum* requires unusual growth conditions. Pus from this sample was tested using Phirst-ID, with results showing an *R*_max_ of 0.989 with *M. marinum*, far higher than for any other species. The highest Pearson correlation coefficient for non-mycobacteria was −0.01 (Supplementary Fig. S9e). Together, these results were strongly suggestive of *M. marinum* infection.

## Discussion

Many current bacterial identification methods rely on culture and biochemical testing. More recent advances, including mass spectrometry and multiplexed PCR, offer progress, with each method improving upon some but not all of the necessary parameters required to transform current clinical microbial diagnostics, namely speed, sensitivity, and/or comprehensiveness. We describe an approach of amplification-free, highly multiplexed fluorescent hybridization to rRNA that enables the rapid identification of a broad range of bacterial pathogens in a single assay from crude lysate, requiring no foreknowledge of the type of bacteria present. We designed and tested the Phirst-ID probeset, a set of 180 probe pairs targeting rRNA sequences from 98 bacterial pathogens, capitalizing on the rich phylogenetic information content, high abundance, and exquisite conservation intrinsic to rRNA sequences. Careful design of probes targeting a mixture of conserved and unique sequences against a broad range of targets overcomes a considerable degree of similarity between closely related species to identify species with >89% accuracy, while also remaining broad enough to classify species across a wide range of bacterial phylogeny in a single assay with 100% accuracy at the family level, which provides useful clinical information. Although the resolution for identifying precise species using Phirst-ID does not yet approach that of MALDI or unbiased sequencing, it can be deployed directly on primary clinical samples and thus return answers faster than these other assays. Further, the scope of pathogens that can be detected using Phirst-ID far exceeds that of targeted multiplexed PCR methods. While we included 180 probes in this implementation, the NanoString assay platform supports the detection of up to 800 RNA targets in a single reaction^30^, leaving room to expand the assay to either broaden the phylogenetic scope or increase resolution in specific cases. By targeting the vast majority of clinically relevant bacterial species, this multiplexed hybridization strategy approaches the utility of a completely unbiased method, identifying major clinically relevant pathogen subsets across a diverse taxonomic range with a significantly simplified, rapid assay directly on primary clinical samples. Moreover, refinement and expansion of the Phirst-ID probeset and of the reference set of strains will enable more accurate pathogen identification. The speed of the assay and its ability to distinguish between closely related sequences promise to be further enhanced by technical developments in the RNA/DNA sequencing and counting assays with the next generation of NanoString instruments (J. Beechem, unpublished observations).

This approach capitalizes on three critical features of rRNA. First, rRNA sequences encode rich phylogenetic information that allow identification of bacteria at multiple taxonomic levels, from species through phylum. By designing a probeset that can query this phylogenetic information to distinguish and identify a broad, diverse set of clinically relevant bacterial pathogens, we have developed a single multiplexed assay that is far faster and easier to perform than genome or amplicon sequencing, and far broader and more unbiased than targeted amplification methods. Second, rRNA is by far the most abundant nucleic acid species in bacteria, present at thousands of copies per cell, allowing high sensitivity without enzymatic amplification. This feature allows signal detection to be made directly from unamplified, crude samples including uncultured clinical samples of sputum, blood cultures diluted 100-fold, and pus. Third, rRNA sequences are nearly invariant within a species and are present in multiple copies per genome in most species, making them less subject to sequence variations across different strains within a species that might compromise the robustness of the assay, as has been seen with certain targeted PCR diagnostics when mutations arise in primer target regions^43,44^. Expanding the probeset to include select mRNA targets could improve specificity and might even enable strain typing within a species. However, given the vastly lower abundance of mRNA compared with rRNA targets, simultaneous detection of both RNA types would exceed the quantitative dynamic range of the assay and reduce sensitivity.

This method for reliable pathogen identification is based on identifying the maximum Pearson correlation coefficient between the Phirst-ID PSRP of the sample of interest and a reference set of known bacterial species. While few single probes encode enough specificity to uniquely identify any individual target species, the aggregate reactivity of the 180 probe pairs is sufficiently unique to identify pathogens to the species level with >89% accuracy in our reference samples, with misassignments being made to closely related species in every case. While certain closely-related taxa such as the *Enterobacteriaceae* family – which are themselves a challenge to current bacterial taxonomy in the genomic era, posing problems for any molecular diagnostic assay^45^ – provide a challenge for species-level identification within the resolution of this assay, this rapid method can confidently classify Gram negatives either into or out of this family, which has considerable clinical consequence for prognostication, empiric antibiotic choice, and informing which clinical breakpoints should be used to interpret antibiotic susceptibility test results^46^. Iterative cycles of probe design targeting hypervariable regions should improve species-level discrimination between closely related species such as the *Enterobacteriaceae*, as will expanding the reference set to include more examples of each species. Because of its phylogeny-informed design, even when testing bacterial species for which we had neither designed nor tested probes, Phirst-ID was able to detect the presence of a bacterium and provide a useful level of phylogenetic information on these rare pathogens based on the isolate in the reference set with the most similar PSRP.

Pilot studies on primary clinical specimens afforded the opportunity to highlight both advantages of this approach and areas of challenge that will need further study. Phirst-ID was able to characterize over 50 primary clinical samples in this pilot study, accurately making pre-specified clinically relevant distinctions in >95% and providing detection and some level of taxonomic information even on samples outside of its reference or design strain sets. As the taxonomic scope of the reference set is expanded through further testing, both against increasing numbers of isolates and new species, the breadth and classification accuracy of Phirst-ID are likely to improve. Iterative cycles of probe design can also expand the scope of the probeset into new phylogenetic areas (e.g., the *Bacteroidetes* phylum encountered in blood culture but not included in design or testing). The advantages of this broad, agnostic approach directly on primary clinical specimens are highlighted by its detection of *M. marinum* in a sample of pus, as this pathogen is extremely difficult to diagnose through standard workflows due to very slow growth in atypical media conditions that require a high degree of pre-test clinical suspicion and considerable microbiology infrastructure. By contrast, Phirst-ID readily identified this pathogen from the primary sample within hours, without the need for culture or any specialized assay conditions. However, as evidenced by several of the clinical sputum samples tested, we recognize that challenges remain with mixed specimens because of the nuanced, non-binary reactivity patterns used to define species. Indeed, mixed samples provide a known challenge for any diagnostic assay that attempts to resolve primary clinical specimens^2^. We anticipate that further computational efforts to identify the bacterial species of simple mixtures from some mixed infections may be possible, as they merely represent the linear combinations of signals from each species present. In this study, we have demonstrated the utility of Phirst-ID on clinical samples, but this sensitive, broad-range assay could have utility in other applications such as agricultural, environmental, or biodefense monitoring. Furthermore, for the moment we have extended the rRNA targeting approach only to bacteria. We previously reported RNA detection for viruses, fungi, and parasites^31^, and given our success in bacteria, rRNA is a reasonable target for enhancing detection sensitivity for all non-viral pathogen types in the near future.

## Methods

### Phirst-ID probeset design

For each of the 98 targeted species (Table S1), all 16S and 23S rRNA sequences were extracted from all representative genomes available from the NCBI RefSeq database^47^ using the Basic Local Alignment Search Tool (BLAST)^48^, comprising a total of 1662 16S and 1933 23S sequences. Within each species, a multiple sequence alignment was constructed to capture any known intra-species diversity. Next, 16S and 23S rRNA sequences were extracted by BLAST from each of the remaining 1122 prokaryotic representative genomes available as of Dec 1, 2015, yielding a total of 3928 16S and 4411 23S sequences to serve as additional outgroups, or in-groups for higher taxonomic levels. Taxonomic classifications for each species were also extracted from the RefSeq database. Having compiled these databases, each desired 16S and 23S target sequence was profiled with NanoString’s probe design algorithm that identifies all putative binding regions for pairs of 50mer probes by considering probe kinetics, secondary structure, and sequence composition^30^. Each putative probe sequence was then aligned using BLAST to our full database of 16S and 23S sequences to identify all potential targets to which hybridization could occur. For each potential probe-pair binding region, a homology score was calculated for each probe to each of its potential hybridization targets. This homology score was used to identify the probe(s) that have the maximum homology score for all intended targets and a minimum homology score for all unintended (cross-hybridization) targets. This analysis was performed at each taxonomic classification level, starting at species level, then progressing to higher levels (genus, family, order, class, and phylum) using the appropriate in- and outgroups for each level (Supplementary Fig. S2). At some classification levels, multiple probes were selected in order to cover the maximum number of intended targets.

### Strain preparation

A total of 117 strains across 64 species were obtained from local hospitals, collaborators, or strain collections (Table S3); where possible, clinical isolates were used. For strains obtained from collaborators or strain collections, strain identification was determined by the provider; for clinical isolates, this was performed using the standard workflow of CLIA certified, clinical microbiology laboratories. Growth conditions are indicated in Table S2; briefly, most strains were grown in liquid media to either mid-log or early stationary phase, then diluted 1:5 in RLT buffer (Qiagen) supplemented with 1% beta-mercaptoethanol (BME). For strains requiring specialized growth conditions, as indicated in Table S3, we directly lysed glycerol stocks obtained from our collaborators by resuspending ~5 uL of frozen material directly in 495 uL of a 1:1 mixture of phosphate-buffered saline (PBS) and RLT buffer with 1% BME. Samples were lysed mechanically via bead-beating for five cycles × one minute on a Minibeadbeater-16 (BioSpec) or one cycle × 90 seconds on a FastPrep (MP Bio) at 10 m/sec. Lysates were either used immediately for hybridization or frozen at −80 °C. For *Mycobacterium tuberculosis* strains, due to biosafety regulations, cultures were prepared in a biosafety-level 3 (BL3) hood, pelleted, and resuspended in Trizol (Life Technologies), then lysed mechanically via bead-beating for 5 cycles × 1 minute on a Minibeadbeater-16 (BioSpec). Chloroform was added prior to removal from the BL3, and the aqueous phase after centrifugation was used directly as the crudest possible lysate for hybridization.

### Quantitative rRNA detection and analysis

rRNA was detected using the Elements assay variation on the standard NanoString assay for multiplexed RNA detection^30^, with several modifications introduced to increase assay speed. Briefly, lysates were diluted in PBS to an estimated 100–300 cells per uL to avoid assay saturation, then 3 uL of this diluted lysate was incubated with unlabeled probe pairs for each target and Elements TagSet-192 reagents^49^. Hybridization conditions were standard, aside from two modifications: lysates were incubated at 95 °C × 2 minutes immediately prior to hybridization to melt secondary structural elements and disrupt protein binding in rRNA targets, and hybridizations were incubated for one hour instead of the recommended 16–24 hours. Hybridizations were then loaded and processed on a Sprint instrument (NanoString) for purification and quantitative detection using a standard run protocol that takes 6.25 hours for a batch of 12 samples, or a modified assay that takes ~2.5 hours for a single sample. Raw count data for each target were read using nSolver software v4.0 (NanoString), then processed as recommended by subtracting the mean of the negative control counts (six probe pairs directed at External RNA Controls Consortium (ERCC) targets not included in the hybridization) and scaling by a factor proportional to the geometric mean of the positive control counts (six probe pairs directed at ERCC spike-ins included in every hybridization at a range of pre-specified concentrations). In addition, eight hybridizations containing PBS buffer alone were run on separate days, and the average of these blank lanes was subtracted from every sample. The resulting normalized, blank-subtracted data was used for all subsequent analysis. All processing from raw counts data, including positive and negative control normalization, blank-subtraction, Pearson correlations, and visualizations were performed in R (version 3.2.3), Python (v 2.7), or Excel (v 16.15). Hierarchical clustering of strains was performed in R using the default (“complete”) method of the hclust() function, using (1 − *R*) as a distance function, where *R* is the Pearson correlation between PSRPs from two strains.

### Clinical sample preparation

15 consecutive primary sputum samples were obtained from the clinical microbiology laboratory at Brigham and Women’s Hospital once their associated culture was found to be positive for a pathogen. Samples were shipped to our laboratory on ice and processed as described^50^; briefly, samples were diluted 10-fold in PBS, sheared through a blunt 14 gauge needle to mechanically disperse, then passed through a 5 um filter (EMD Millipore), then mixed 1:1 with RLT buffer with 1% BME. 37 consecutive positive blood culture bottles from unique patients were collected from the clinical microbiology laboratory at Massachusetts General Hospital. An aliquot was spun at 100 × g for 10 minutes to sediment RBCs and other large debris, then 100 uL of supernatant was added to 400 uL of RLT buffer (Qiagen) +1% BME. One sample was collected from a laboratory worker who volunteered pus for the assay after an exposure to *Mycobacterium marinum*. The worker was independently treated elsewhere for this infection. Spontaneously draining pus was sampled with a sterile cotton swab and resuspended in 100 uL of RLT buffer with 1% BME. All clinical samples were then lysed mechanically via bead-beating for one 90-second cycle on a FastPrep instrument (MP Bio) at 10 m/sec. Blood culture samples were diluted an additional 20x in PBS prior to hybridization to avoid saturation in the NanoString detection assay. Lysates were either used immediately or frozen at −80 °C. Researchers remained blinded to all clinical culture results until after an identification was made using the probeset.

### Ethical approval and informed consent

Discarded clinical samples from Massachusetts General Hospital and Brigham and Women’s Hospital were obtained under waiver of consent due to exclusive focus on pathogen and not host contents, as approved by the Partners Health Care Institutional Review Board that governs both institutions, under protocol number 2015P002215. Results from these investigational studies were not released to the clinical care providers, but were compared to results from standard clinical workflows.

## Supplementary information

Supplementary Information

Supplementary Table S1

Supplementary Table S2

Supplementary Table S3

## Data Availability

The datasets used and/or analyzed during the current study are available from the corresponding authors on reasonable request.
