# Supplementary material for: Rapid identification and phylogenetic classification of diverse bacterial pathogens in a multiplexed hybridization assay targeting ribosomal RNA
Source: Sci Rep. 2019 Mar 14;9:4516. doi: 10.1038/s41598-019-40792-3 (PMC6418090; doi:10.1038/s41598-019-40792-3)
Supplement: Supplementary file 1 — Supplementary Information [file 41598_2019_40792_MOESM1_ESM.pdf]

Supplementary Information

Rapid identification and phylogenetic classification of diverse bacterial pathogens in a multiplexed hybridization assay targeting ribosomal RNA

Roby P. Bhattacharyya[1,2], Mark Walker[1], Rich Boykin[3], Sophie S. Son[1], Jamin Liu[1,4], Austin C. Hachey[1,5], Peijun Ma[1], Lidan Wu[1,6], Kyungyong Choi[7], Kaelyn C. Cummins[8,9], Maura Benson[8], Jennifer Skerry[10], Hyunryul Ryu[7,11], Sharon Y. Wong[1], Marcia B. Goldberg[2], Jongyoon Han[7,12], Virginia M. Pierce[10], Lisa A. Cosimi[8], Noam Shoresh[1], Jonathan Livny[1], Joseph Beechem*[3], Deborah T. Hung*[1,13,14]

[1] Infectious Disease and Microbiome Program, Broad Institute of Harvard and MIT, 415 Main St, Cambridge MA 02142

[2] Infectious Diseases Division, Department of Medicine, Massachusetts General Hospital, 55 Fruit St, Boston MA 02114

[3] NanoString Technologies, Inc., 530 Fairview Ave N, Seattle WA 98109

[4] Present address: UC Berkeley – UCSF Graduate Program in Bioengineering, Department of Bioengineering and Therapeutic Sciences, 1700 4th St, San Francisco CA 94158

[5] Present address: Department of Chemistry, College of Arts & Sciences, University of Kentucky, Lexington KY 40506

[6] Present address: NanoString Technologies, Inc., 530 Fairview Avenue N, Seattle WA 98109

[7] Research Laboratory of Electronics and Department of Electrical Engineering and Computer Science, Massachusetts Institute of Technology, 77 Massachusetts Ave, Cambridge MA 02139

[8] Infectious Diseases Division, Department of Medicine, Brigham and Women's Hospital, 75 Francis St, Boston MA 02115

[9] Present address: Baylor College of Medicine, 1 Baylor Plaza, Houston TX 77030

[10] Microbiology Laboratory, Department of Pathology, Massachusetts General Hospital, 55 Fruit St, Boston MA 02114

[11] Present address: Twoporeguys, Inc., 2155 Delaware Ave, Santa Cruz CA 95060

[12] Department of Biological Engineering, Massachusetts Institute of Technology, 77 Massachusetts Ave, Cambridge MA 02139

[13] Department of Genetics, Harvard Medical School, 77 Ave Louis Pasteur, Boston MA, 02115

[14] Department of Molecular Biology and Center for Computational and Integrative Biology, Massachusetts General Hospital, 185 Cambridge Street, Boston MA, 02114

*Co-corresponding authors: jbeechem@nanostring.com; hung@molbio.mgh.harvard.edu

Supplementary Figure Legends:

Figure S1: Target regions of 180 Phirst-ID probes on 16S and 23S rRNA subunits. Each of the 180 probes in the Phirst-ID probeset is shown, in the same order as **Fig. 1c** and **Table S1**, as it maps onto a consensus alignment of the 16S or 23S subunits built from all 98 species targeted in probe design. Apparent gaps in probes are the result of insertions from one or more of the 98 targets causing extension of the consensus alignment; all probes hybridize to contiguous regions in their cognate species targets. Colored dot next to each probe indicates intended taxonomic level.

Figure S2: Schematic of Phirst-ID probeset design strategy. Conceptual schematic of ingroup (blue) and outgroup (orange) selection for hierarchical probe design strategy. Species being deliberately targeted for design (98 total species in this manuscript) are represented in bold font; species not targeted for design but considered for the purposes of cross-hybridization (1122 total species in this manuscript) are represented in italics. **(a)** For species-level probe design, only the targeted species (Sp1) is considered the ingroup; all remaining species are outgroups. **(b)** For genus-level probe design, all members of the targeted genus (Gen1) are considered ingroups, while all remaining species are outgroups. This strategy is extrapolated stepwise to progressively higher levels of phylogeny (family, order, class, phylum).

Figure S3: Replicates are well correlated. Normalized probe intensities and correlations from a representative example of **(a)** technical and **(b)** biological replicates from a *Staphylococcus aureus* species.

Figure S4: Pairwise correlation coefficients from PSRPs reflect taxonomic relationships between reference strains. Heatmap of pairwise Pearson correlation coefficients (*R* values)

between all 117 strains in the reference set. Strains are shown in the same order as **Fig. 1**, with taxonomic classifications shown above. Strain order is identical on the horizontal and vertical axes; self-correlations are included on the diagonal. Notable taxonomic classifications are labeled along the diagonal.

Figure S5: Maximum pairwise Pearson correlation coefficient (R_{max}) identifies isolates with increasing accuracy at higher phylogenetic levels. For each indicated taxonomic classification level, species **(a)** through phylum **(f)**, the pairwise R_{max} for a member of the same taxonomic classification level is plotted on the x-axis, and the pairwise R_{max} for all other strains NOT in that taxonomic classification level is plotted on the y-axis. Blue = data from strains for which multiple isolates were tested; red = data from species for which a single isolate was tested. Points below the diagonal line (y = x) represent correct strain identifications at each taxonomic level, with tabulated success rates shown on each plot. Distance from the diagonal reflects confidence of classification.

Figure S6: Pairwise Pearson correlation coefficients for species tested without replicates identify closest possible taxonomic match. All 116 non-self pairwise Pearson correlation coefficients (R) plotted for each of the 42 species for which only one isolate was obtained for testing, colored by taxonomic relationship of the paired species. Order of these species is the same as **Fig. 1a** (white boxes only).

Figure S7: Probes targeting taxonomic classes represented by clinically relevant subsets help to distinguish amongst these species. Normalized intensity data from the full subset of probes designed to target all **(a)** *Staphylococcus*, **(b)** *Mycobacterium*, **(c)** *Enterococcus*, and **(d)** *Gammaproteobacteria* species in the Phirst-ID design, tested against each member of their respective taxa from the reference strain set. Taxonomic level of each probe indicated by color

scale at right. Differences in reactivity amongst these probes underlie most of the differences in R_{max} between these species seen in **Fig. 3**.

Figure S8: PSRPs of untargeted species reflect nearest taxonomic relatives. (a) Heatmap of pairwise Pearson correlation coefficients between six “untargeted” isolates (i.e., species not included in the design process) from four species in the reference set (horizontal axis), against the remaining 111 isolates from the reference set (vertical axis). Best matches among the targeted strains in the reference set, along with corresponding R_{max}, listed at right. **(b)** PSRPs for each untargeted isolate (red species labels) and their nearest targeted neighbors from the reference set (black species labels). Select probe targets are shown at right for each set of isolates; black indicates an “on-target” or intended match, gray indicates an “off-target” match i.e. cross-reactivity.

Figure S9: PSRPs of clinical samples reveal causal pathogens: (a) Heatmap of pairwise Pearson correlations from PSRPs of 15 clinical sputum samples against the 117-strain reference set. Top panel shows success of identification based on these correlations. **(b)** PSRPs from selected sputum samples with mixed organisms on Gram stain shown as normalized intensity data from all Phirst-ID probes. **(c)** Heatmap of pairwise *R* values for 37 blood culture samples against the 117-strain reference set. **(d)** PSRPs from the five blood cultures that grew organisms not targeted below the family level in either probeset design or the reference set of strains. *Corynebacterium* and *D. hominis* are in the phylum *Actinobacteria*; the two *Bacteroides* species are in the phylum *Bacteroidetes*. **(e)** Heatmap of pairwise *R* values for pus sample against the 117-strain reference set.

Figure S1

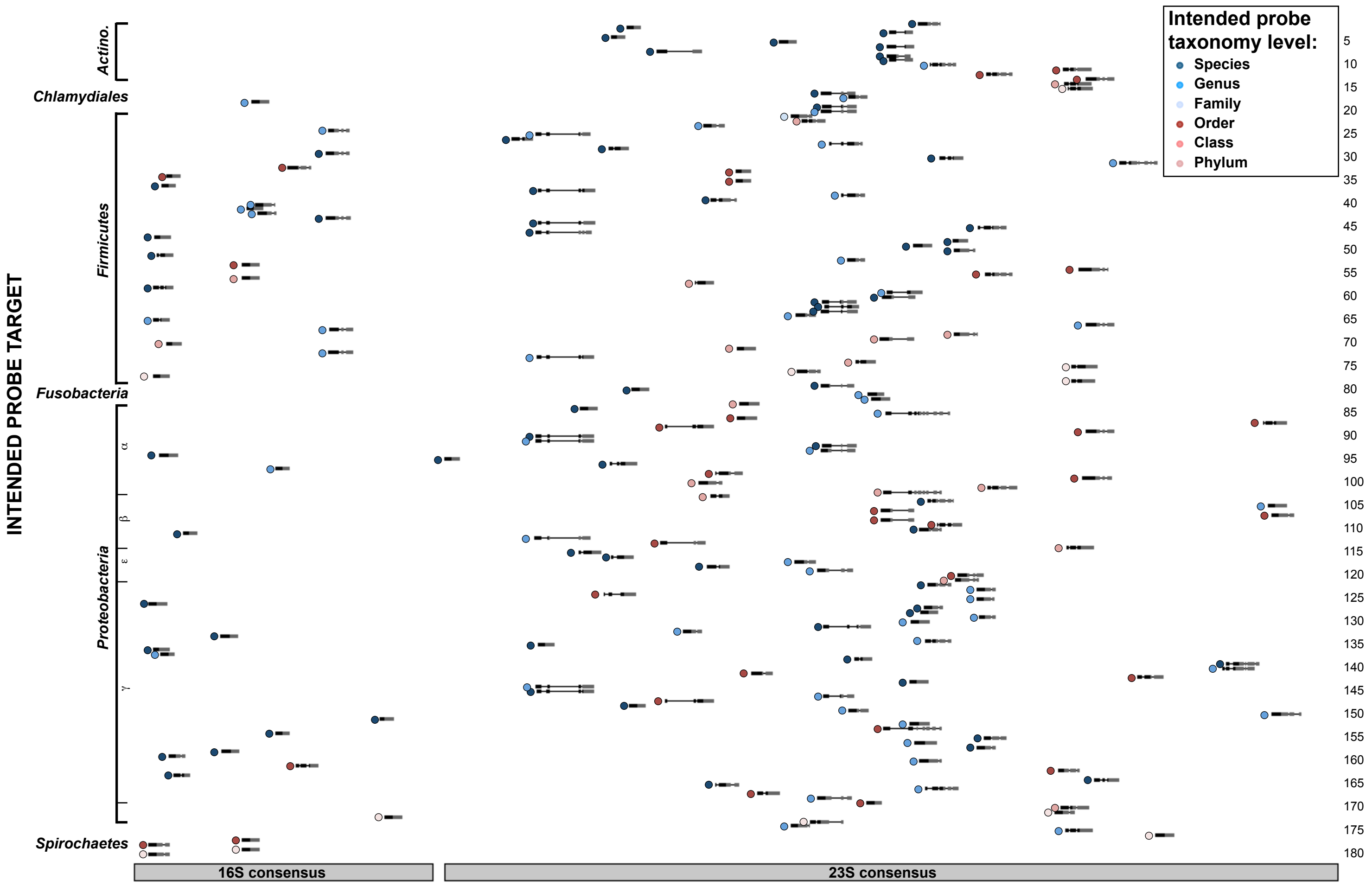

INTENDED PROBE TARGET
Actino.
Chlamydiales
Firmicutes
Fusobacteria
Proteobacteria
α
β
ε
γ
Spirochaetes
Intended probe taxonomy level:
Species
Genus
Family
Order
Class
Phylum
5
10
15
20
25
30
35
40
45
50
55
60
65
70
75
80
85
90
95
100
105
110
115
120
125
130
135
140
145
150
155
160
165
170
175
180
16S consensus
23S consensus

Figure S2

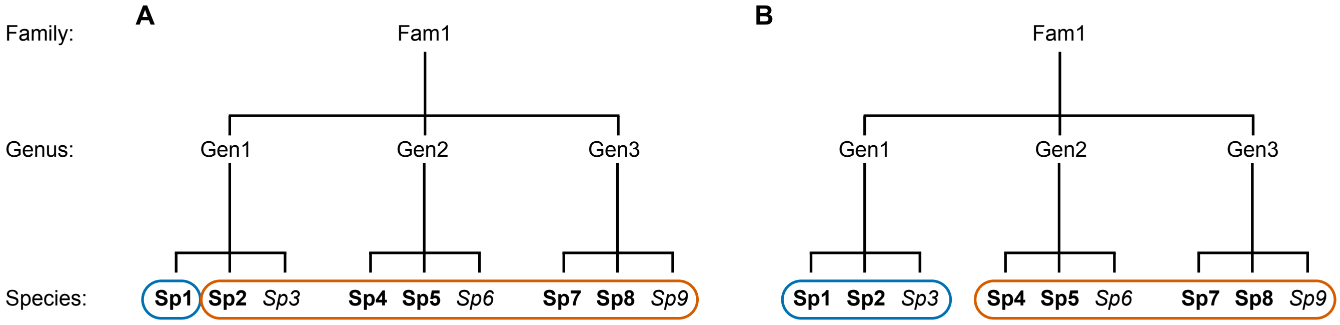

Family:
Genus:
Species:
A
Fam1
Gen1
Gen2
Gen3
Sp1
Sp2
Sp3
Sp4
Sp5
Sp6
Sp7
Sp8
Sp9
B
Fam1
Gen1
Gen2
Gen3
Sp1
Sp2
Sp3
Sp4
Sp5
Sp6
Sp7
Sp8
Sp9

Figure S3

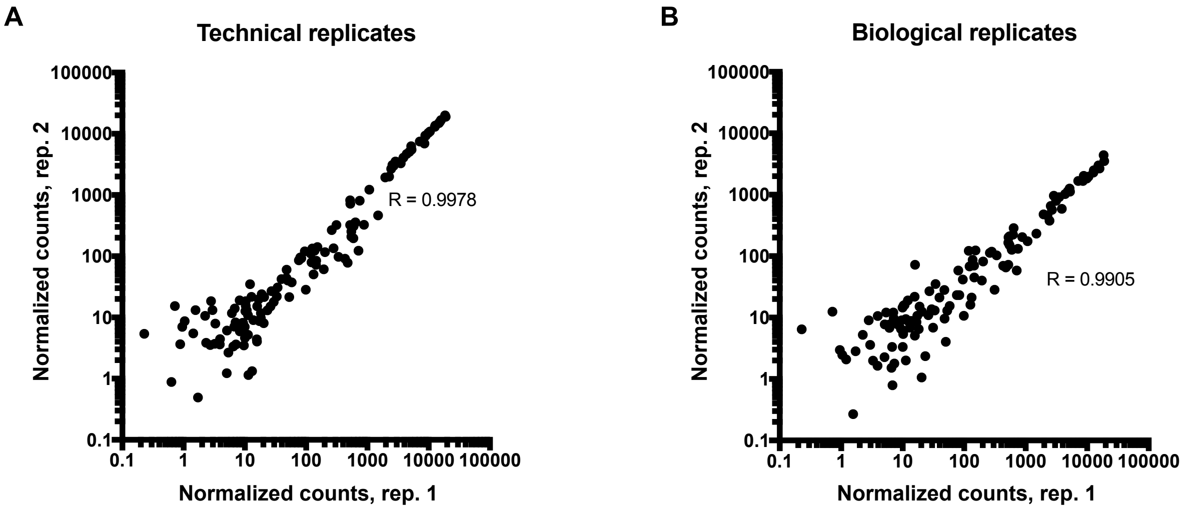

A
Technical replicates
Normalized counts, rep. 2
Normalized counts, rep. 1
R = 0.9978
100000
10000
1000
100
10
1
0.1
0.1
1
10
100
1000
10000
100000
B
Biological replicates
Normalized counts, rep. 2
Normalized counts, rep. 1
R = 0.9905

Figure S4

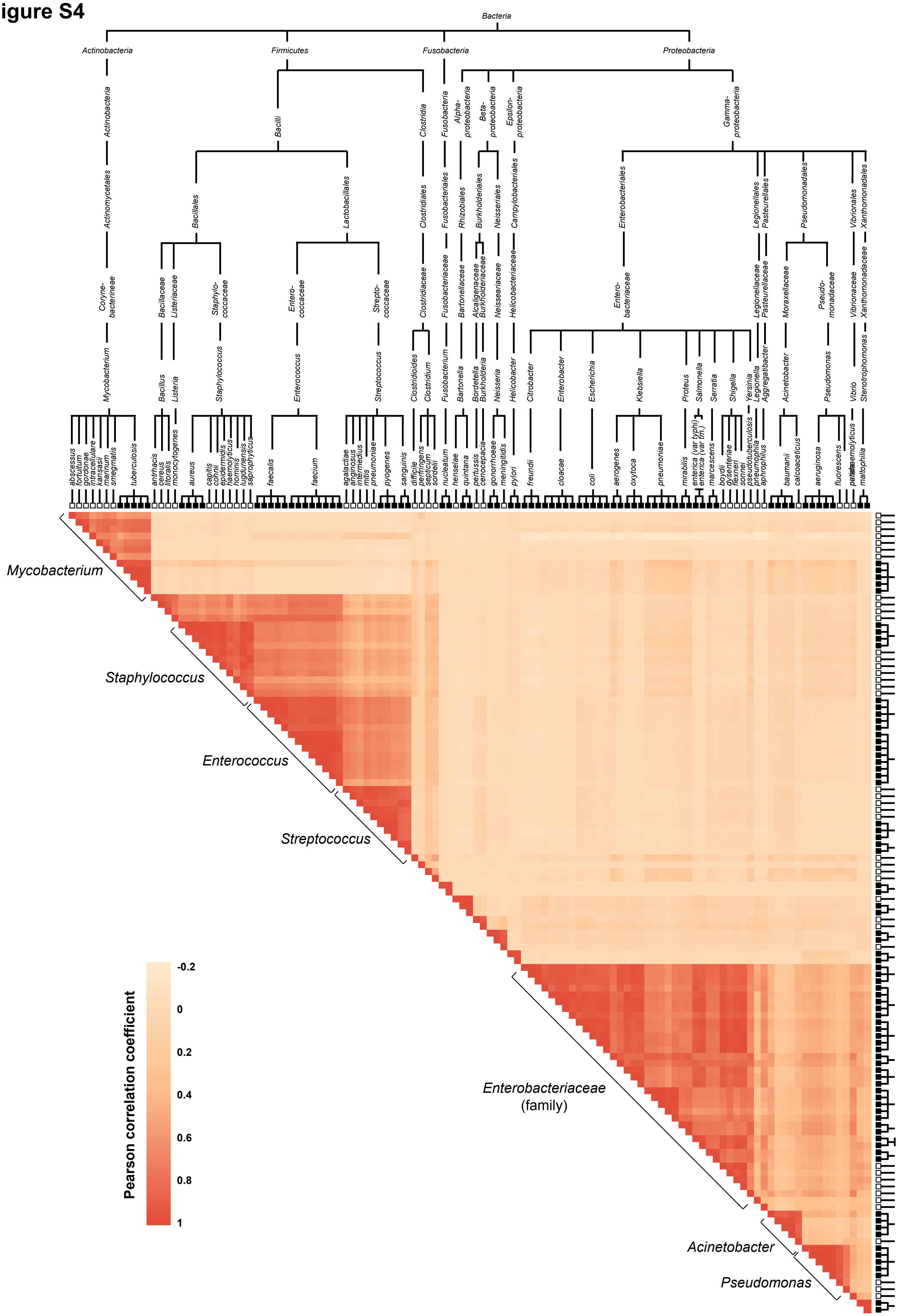

Bacteria
Actinobacteria
Firmicutes
Fusobacteria
Proteobacteria
Actinobacteria
Bacilli
Clostridia
Fusobacteria
Alpha-proteobacteria
Beta-proteobacteria
Epsilon-proteobacteria
Gamma-proteobacteria
Actinomycetales
Bacillales
Lactobacillales
Clostridiales
Fusobacteriales
Rhizobiales
Burkholderiales
Neisseriales
Campylobacteriales
Enterobacteriales
Legionellales
Pasteurellales
Pseudomonadales
Vibrionales
Xanthomonadales
Coryne-bacterineae
Bacillaceae
Listeriaceae
Staphylo-coccaceae
Entero-coccaceae
Strepto-coccaceae
Clostridiaceae
Fusobacteriaceae
Bartonellaceae
Alcaligenaceae
Burkholderiaceae
Neisseriaceae
Helicobacteriaceae
Entero-bacteriaceae
Legionellaceae
Pasteurellaceae
Moraxellaceae
Pseudo-monadaceae
Vibrionaceae
Xanthomonadaceae
Mycobacterium
Bacillus
Listeria
Staphylococcus
Enterococcus
Streptococcus
Clostridioides
Clostridium
Fusobacterium
Bartonella
Bordetella
Burkholderia
Neisseria
Helicobacter
Citrobacter
Enterobacter
Escherichia
Klebsiella
Proteus
Salmonella
Serratia
Shigella
Yersinia
Legionella
Aggregatibacter
Acinetobacter
Pseudomonas
Vibrio
Stenotrophomonas
abscessus
fortuitum
gordonae
intracellulare
kansasii
marinum
smegmatis
tuberculosis
anthracis
cereus
litoralis
monocytogenes
aureus
capitis
cohnii
epidermidis
haemolyticus
hominis
lugdunensis
saprophyticus
faecalis
faecium
agalactiae
anginosus
intermedius
mitis
pneumoniae
pyogenes
sanguinis
difficile
perfringens
septicum
sordellii
nucleatum
henselae
quintana
pertussis
cenocepacia
gonorrhoeae
meningitidis
pylori
freundii
cloacae
coli
aerogenes
oxytoca
pneumoniae
mirabilis
enterica (var typhi)
enterica (var tm.)
marcescens
boydii
dysenteriae
flexneri
sonnei
pseudotuberculosis
pneumophila
aphrophilus
baumanii
calcoaceticus
aeruginosa
fluorescens
parahaemolyticus
maltophilia
Mycobacterium
Staphylococcus
Enterococcus
Streptococcus
Enterobacteriaceae
(family)
Acinetobacter
Pseudomonas
Pearson correlation coefficient
-0.2
0
0.2
0.4
0.6
0.8
1

Figure S5

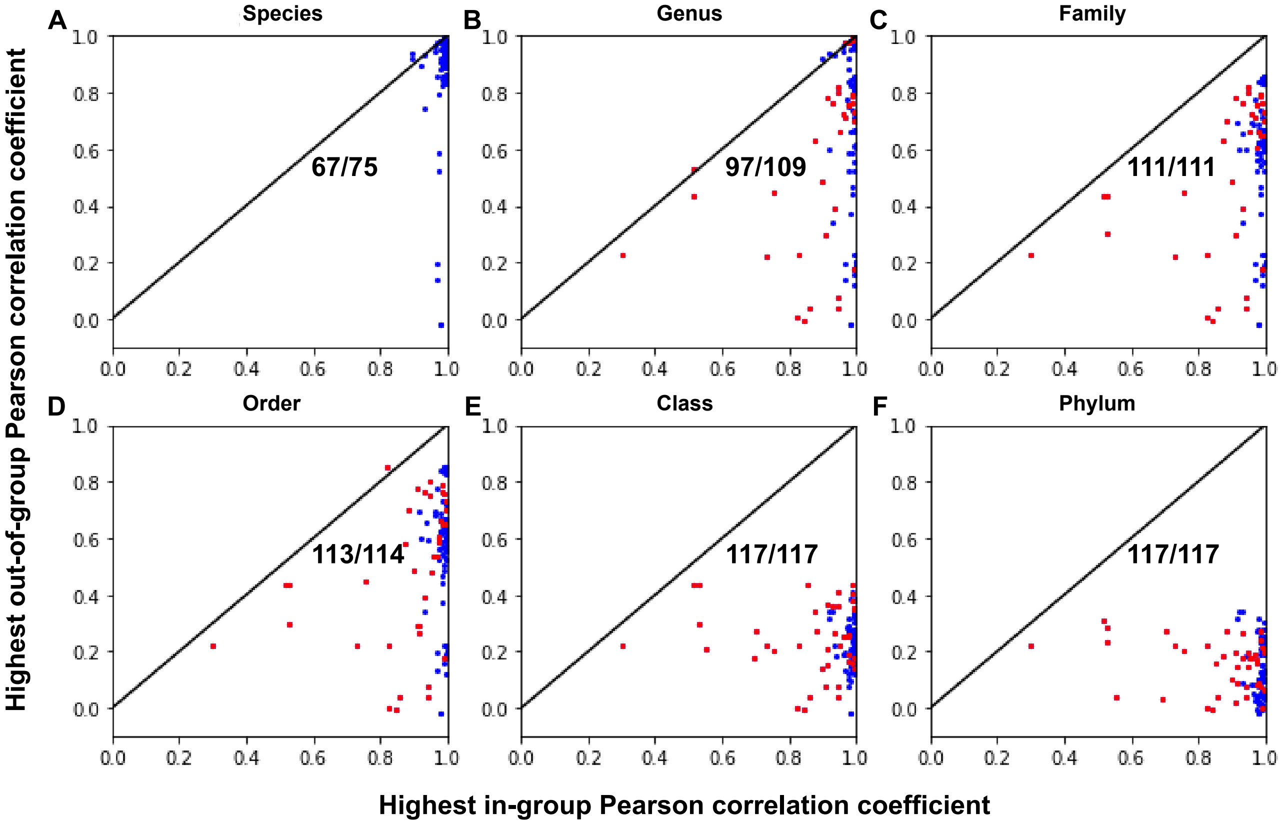
A
Species
67/75
B
Genus
97/109
C
Family
111/111
D
Order
113/114
E
Class
117/117
F
Phylum
117/117
Highest out-of-group Pearson correlation coefficient
Highest in-group Pearson correlation coefficient
0.0
0.2
0.4
0.6
0.8
1.0

Figure S6

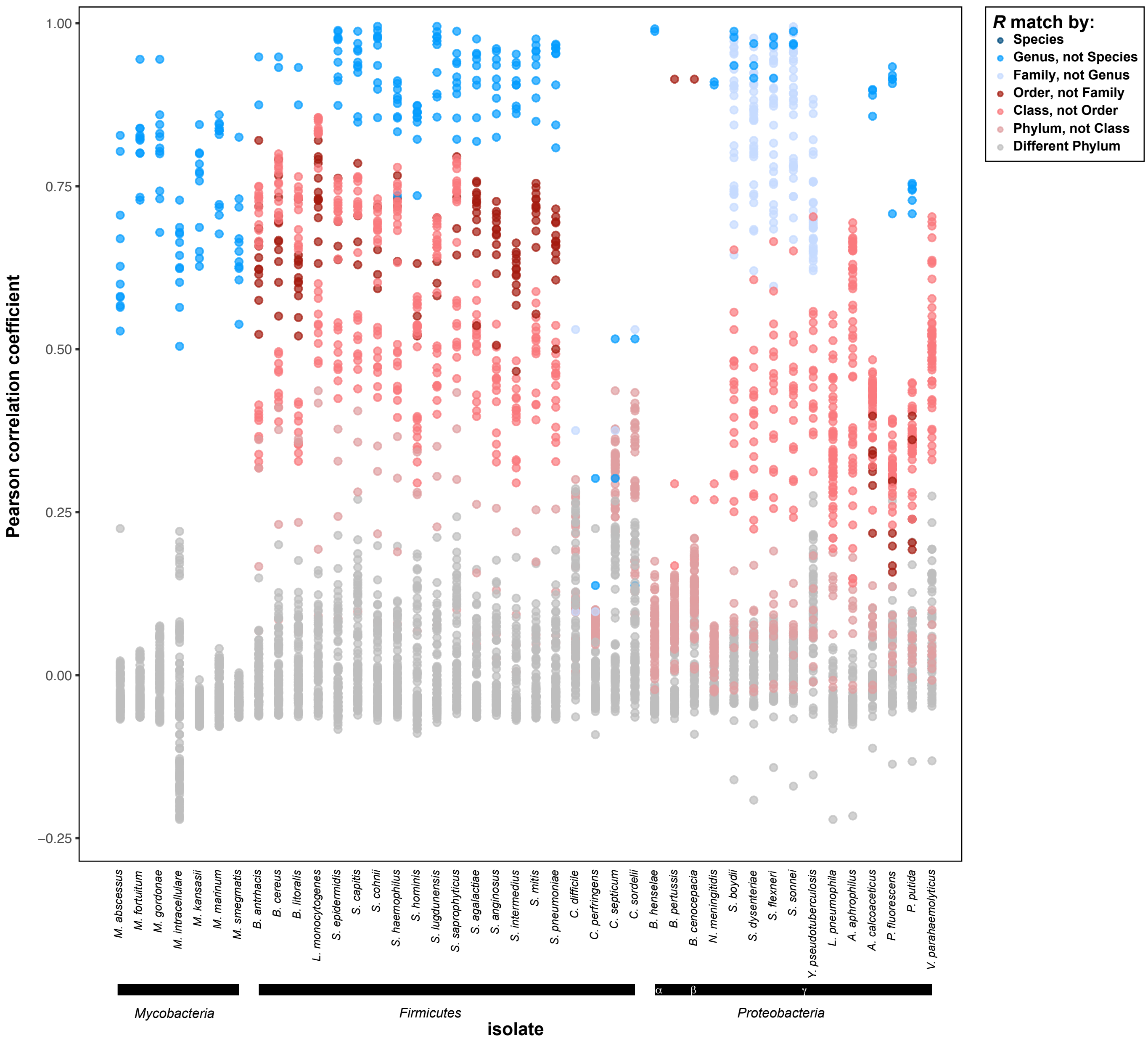

Pearson correlation coefficient
1.00
0.75
0.50
0.25
0.00
−0.25
R match by:
Species
Genus, not Species
Family, not Genus
Order, not Family
Class, not Order
Phylum, not Class
Different Phylum
M. abscessus
M. fortuitum
M. gordonae
M. intracellulare
M. kansasii
M. marinum
M. smegmatis
B. anthracis
B. cereus
B. litoralis
L. monocytogenes
S. epidermidis
S. capitis
S. cohnii
S. haemophilus
S. hominis
S. lugdunensis
S. saprophyticus
S. agalactiae
S. anginosus
S. intermedius
S. mitis
S. pneumoniae
C. difficile
C. perfringens
C. septicum
C. sordelii
B. henselae
B. pertussis
B. cenocepacia
N. meningitidis
S. boydii
S. dysenteriae
S. flexneri
S. sonnei
Y. pseudotuberculosis
L. pneumophila
A. aphrophilus
A. calcoaceticus
P. fluorescens
P. putida
V. parahaemolyticus
α
β
γ
Mycobacteria
Firmicutes
Proteobacteria
isolate

Figure S7

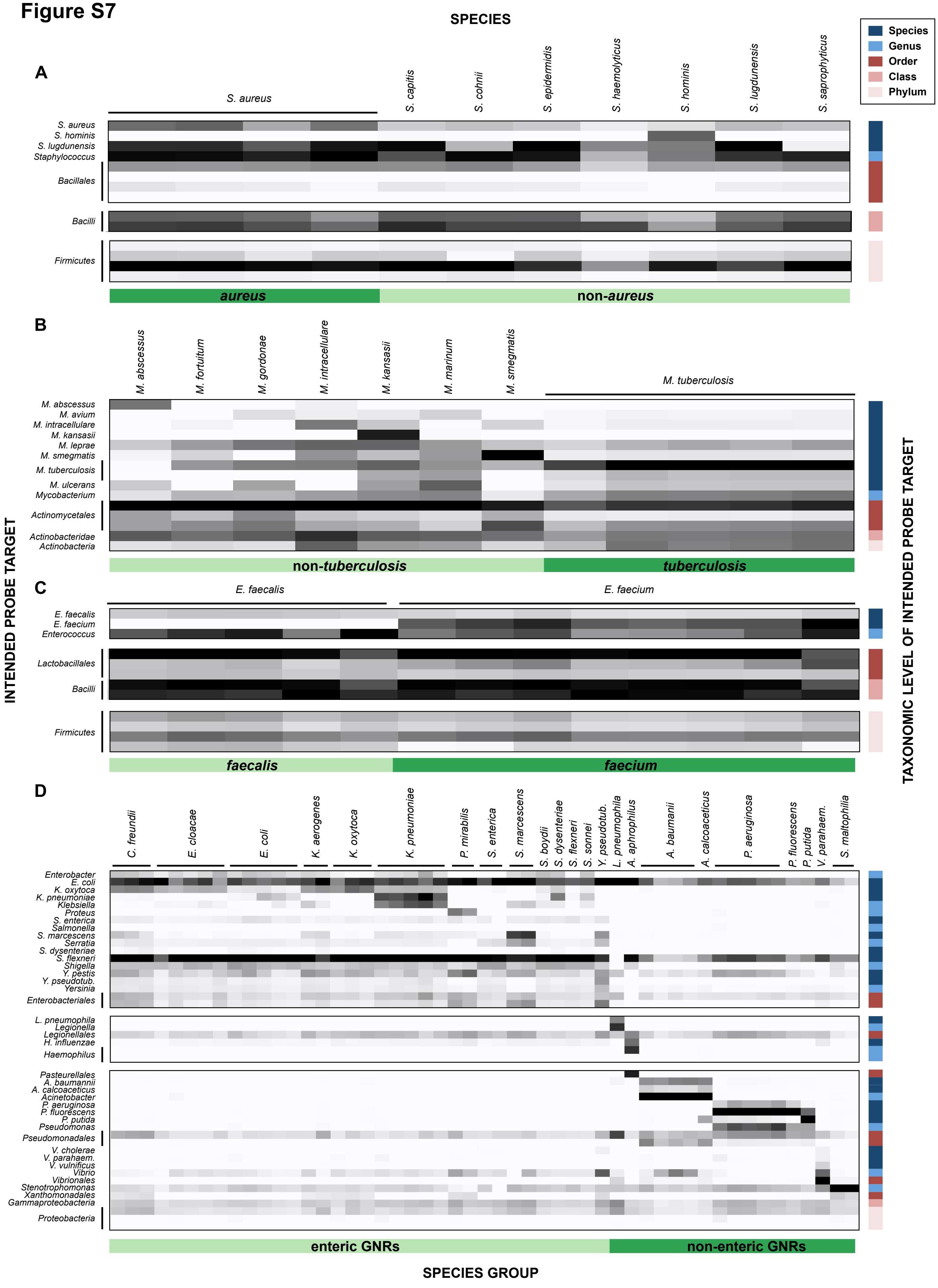
SPECIES
Species
Genus
Order
Class
Phylum
A
S. aureus
S. capitis
S. cohnii
S. epidermidis
S. haemolyticus
S. hominis
S. lugdunensis
S. saprophyticus
S. aureus
S. hominis
S. lugdunensis
Staphylococcus
Bacillales
Bacilli
Firmicutes
aureus
non-aureus
B
M. abscessus
M. fortuitum
M. gordonae
M. intracellulare
M. kansasii
M. marinum
M. smegmatis
M. tuberculosis
M. abscessus
M. avium
M. intracellulare
M. kansasii
M. leprae
M. smegmatis
M. tuberculosis
M. ulcerans
Mycobacterium
Actinomycetales
Actinobacteridae
Actinobacteria
non-tuberculosis
tuberculosis
C
E. faecalis
E. faecium
E. faecalis
E. faecium
Enterococcus
Lactobacillales
Bacilli
Firmicutes
faecalis
faecium
D
C. freundii
E. cloacae
E. coli
K. aerogenes
K. oxytoca
K. pneumoniae
P. mirabilis
S. enterica
S. marcescens
S. boydii
S. dysenteriae
S. flexneri
S. sonnei
Y. pseudotub.
L. pneumophila
A. aphrophilus
A. baumanii
A. calcoaceticus
P. aeruginosa
P. fluorescens
P. putida
V. parahaem.
S. maltophilia
Enterobacter
E. coli
K. oxytoca
K. pneumoniae
Klebsiella
Proteus
S. enterica
Salmonella
S. marcescens
Serratia
S. dysenteriae
S. flexneri
Shigella
Y. pestis
Y. pseudotub.
Yersinia
Enterobacteriales
L. pneumophila
Legionella
Legionellales
H. influenzae
Haemophilus
Pasteurellales
A. baumannii
A. calcoaceticus
Acinetobacter
P. aeruginosa
P. fluorescens
P. putida
Pseudomonas
Pseudomonadales
V. cholerae
V. parahaem.
V. vulnificus
Vibrio
Vibrionales
Stenotrophomonas
Xanthomonadales
Gammaproteobacteria
Proteobacteria
enteric GNRs
non-enteric GNRs
SPECIES GROUP
INTENDED PROBE TARGET
TAXONOMIC LEVEL OF INTENDED PROBE TARGET

Figure S8

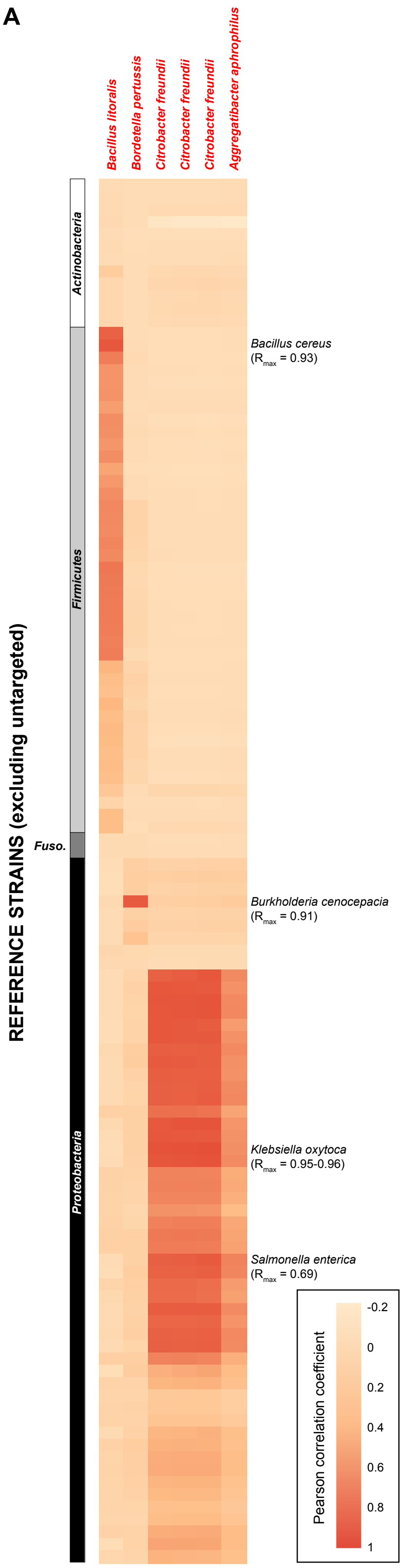

A
REFERENCE STRAINS (excluding untargeted)
Bacillus litoralis
Bordetella pertussis
Citrobacter freundii
Citrobacter freundii
Citrobacter freundii
Aggregatibacter aphrophilus
Actinobacteria
Firmicutes
Fuso.
Proteobacteria
Bacillus cereus
(R$_{max}$ = 0.93)
Burkholderia cenocepacia
(R$_{max}$ = 0.91)
Klebsiella oxytoca
(R$_{max}$ = 0.95-0.96)
Salmonella enterica
(R$_{max}$ = 0.69)
Pearson correlation coefficient
-0.2
0
0.2
0.4
0.6
0.8
1

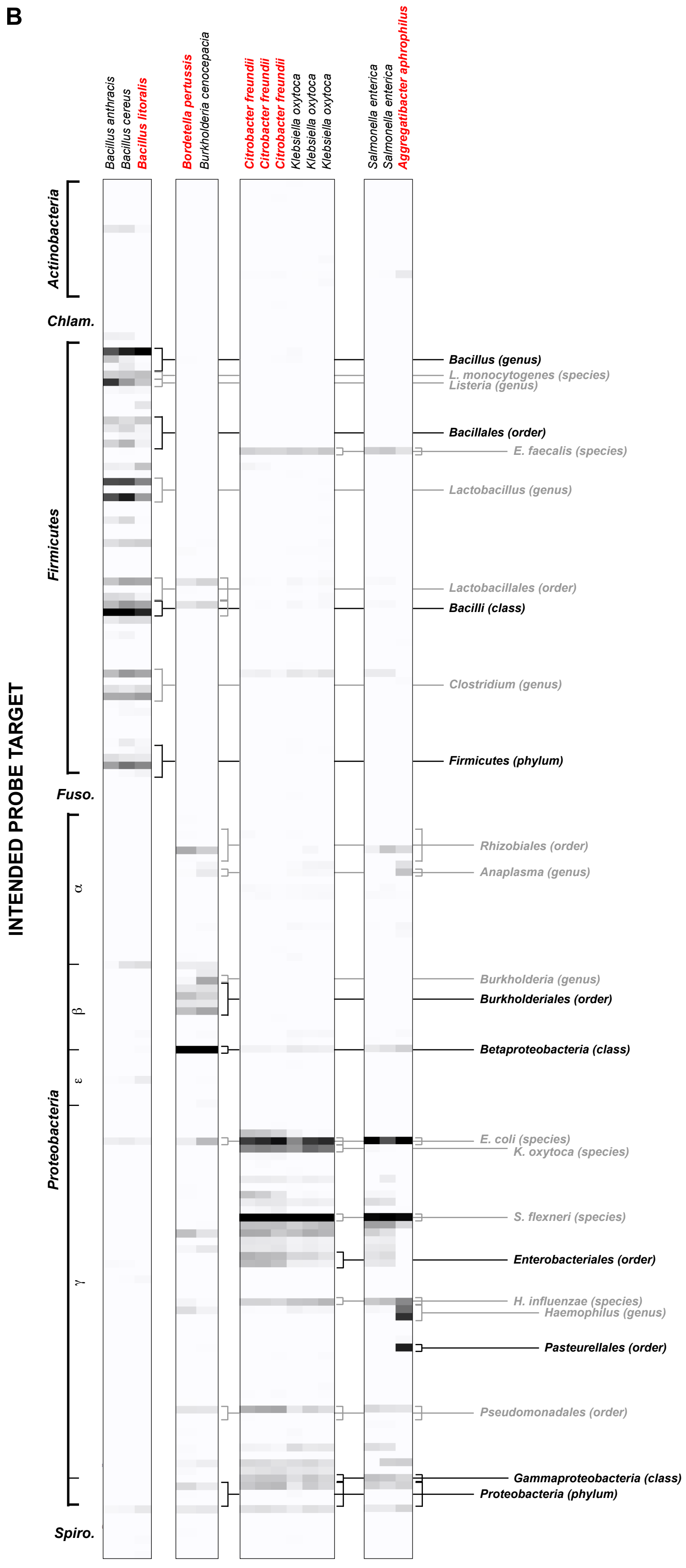

B
INTENDED PROBE TARGET
Bacillus anthracis
Bacillus cereus
Bacillus litoralis
Bordetella pertussis
Burkholderia cenocepacia
Citrobacter freundii
Citrobacter freundii
Citrobacter freundii
Klebsiella oxytoca
Klebsiella oxytoca
Klebsiella oxytoca
Salmonella enterica
Salmonella enterica
Aggregatibacter aphrophilus
Actinobacteria
Chlam.
Firmicutes
Fuso.
Proteobacteria
α
β
ε
γ
Spiro.
Bacillus (genus)
L. monocytogenes (species)
Listeria (genus)
Bacillales (order)
E. faecalis (species)
Lactobacillus (genus)
Lactobacillales (order)
Bacilli (class)
Clostridium (genus)
Firmicutes (phylum)
Rhizobiales (order)
Anaplasma (genus)
Burkholderia (genus)
Burkholderiales (order)
Betaproteobacteria (class)
E. coli (species)
K. oxytoca (species)
S. flexneri (species)
Enterobacteriales (order)
H. influenzae (species)
Haemophilus (genus)
Pasteurellales (order)
Pseudomonadales (order)
Gammaproteobacteria (class)
Proteobacteria (phylum)

Figure S9

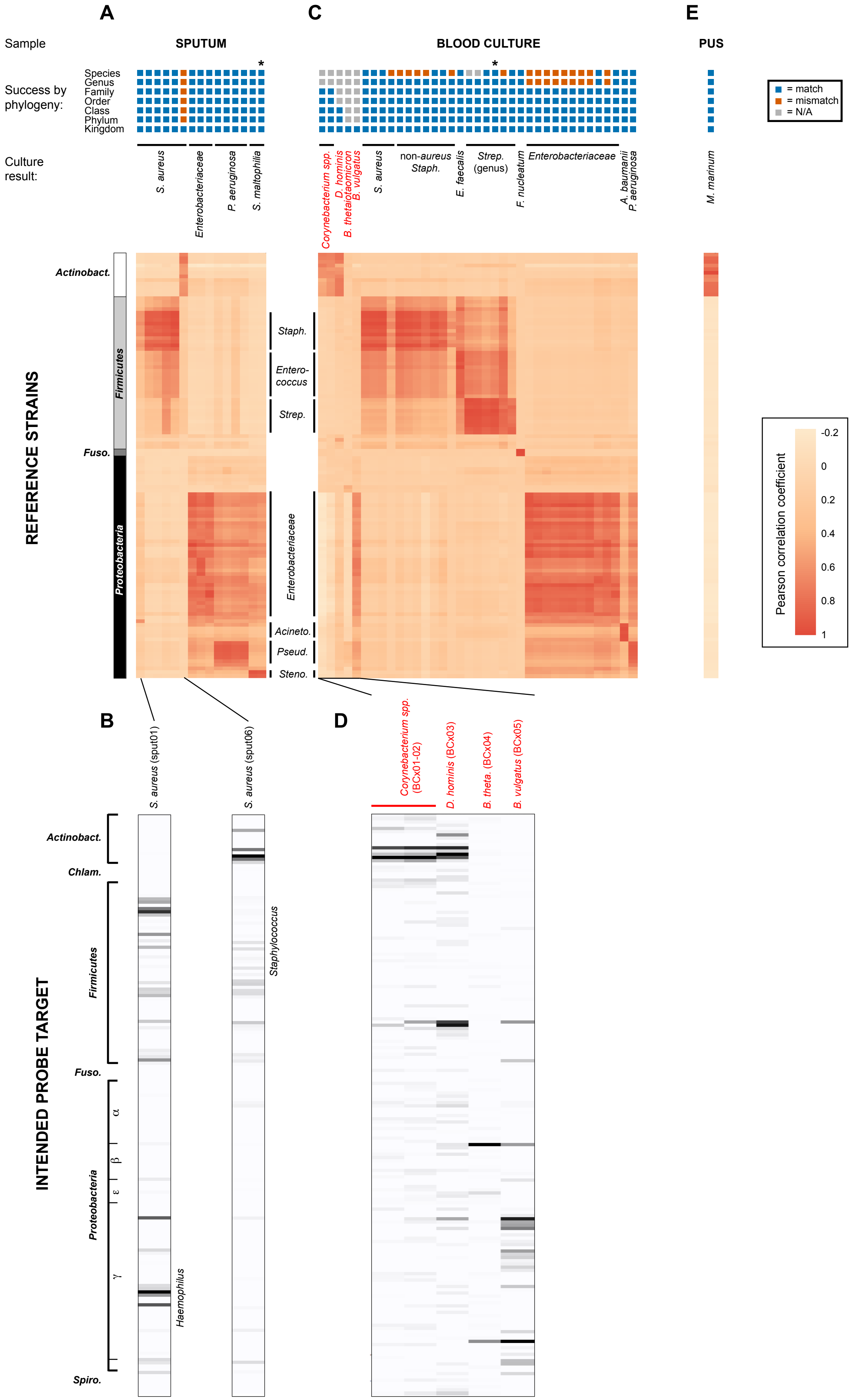

A
C
E
Sample
SPUTUM
BLOOD CULTURE
PUS
Success by phylogeny:
Species
Genus
Family
Order
Class
Phylum
Kingdom
= match
= mismatch
= N/A
Culture result:
S. aureus
Enterobacteriaceae
P. aeruginosa
S. maltophilia
Corynebacterium spp.
D. hominis
B. thetaiotaomicron
B. vulgatus
S. aureus
non-aureus Staph.
E. faecalis
Strep. (genus)
F. nucleatum
Enterobacteriaceae
A. baumanii
P. aeruginosa
M. marinum
REFERENCE STRAINS
Actinobact.
Firmicutes
Fuso.
Proteobacteria
Staph.
Entero-coccus
Strep.
Enterobacteriaceae
Acineto.
Pseud.
Steno.
Pearson correlation coefficient
-0.2
0
0.2
0.4
0.6
0.8
1
B
D
S. aureus (sput01)
S. aureus (sput06)
Corynebacterium spp. (BCx01-02)
D. hominis (BCx03)
B. theta. (BCx04)
B. vulgatus (BCx05)
INTENDED PROBE TARGET
Actinobact.
Chlam.
Firmicutes
Fuso.
Proteobacteria
α
β
ε
γ
Spiro.
Staphylococcus
Haemophilus
